# Supplementary material for: Time-series cardiovascular risk factors and receipt of screening for breast, cervical, and colon cancer: The Guideline Advantage
Source: PLoS One. 2020 Aug 13;15(8):e0236836. doi: 10.1371/journal.pone.0236836 (PMC7425922; doi:10.1371/journal.pone.0236836)
Supplement: Attachment — Submitted filename: Response to Reviewers PONE-D-20-10777R1.docx [file pone.0236836.s001.docx]

**Response to reviewer comments**

We would like to thank reviewers for the helpful comments. Please find our responses to the reviews below in bold. We hope you find the revised manuscript suitable for publication.

**Journal Requirements:**

When submitting your revision, we need you to address these additional requirements.

1. Please ensure that your manuscript meets PLOS ONE's style requirements, including those for file naming. The PLOS ONE style templates can be found at

<https://journals.plos.org/plosone/s/file?id=wjVg/PLOSOne_formatting_sample_main_body.pdf> and

<https://journals.plos.org/plosone/s/file?id=ba62/PLOSOne_formatting_sample_title_authors_affiliations.pdf>

**Thank you for the instructions, we have modified the manuscript according to the instructions.**

2. In ethics statement in the manuscript and in the online submission form, please provide additional information about the patient records used in your retrospective study. Specifically, please ensure that you have discussed whether all data were fully anonymized before you accessed them and/or whether the IRB or ethics committee waived the requirement for informed consent. If patients provided informed written consent to have data from their medical records used in research, please include this information.

**Thank you. We have added the details “All the data were fully anonymized before we accessed them.”**

3. We note that you have stated that you will provide repository information for your data at acceptance. Should your manuscript be accepted for publication, we will hold it until you provide the relevant accession numbers or DOIs necessary to access your data. If you wish to make changes to your Data Availability statement, please describe these changes in your cover letter and we will update your Data Availability statement to reflect the information you provide.

**Thank you. In our Data Availability statement, we stated that we will be able to share the Python code related to the analyses at the time of acceptance of the manuscript. As the data owned by a third party, we do not have permission to share the data. Requesting access to The Guideline Advantage (TGA) data must be requested from the American Heart Association.**

4. Your ethics statement must appear in the Methods section of your manuscript. If your ethics statement is written in any section besides the Methods, please move it to the Methods section and delete it from any other section. Please also ensure that your ethics statement is included in your manuscript, as the ethics section of your online submission will not be published alongside your manuscript.

**Thank you. We have moved the ethics statement to the beginning of the Methods section.**

5. We note you have included a table to which you do not refer in the text of your manuscript. Please ensure that you refer to Table 2 in your text; if accepted, production will need this reference to link the reader to the Table.

**Thank you for the great comment. We have included the Table 2 in the text.**

6. Please include a caption for figure 3.

**Thank you. We have corrected figure 3 to figure 2 which included a caption.**

Reviewers' comments:

Reviewer's Responses to Questions

**Comments to the Author**

1. Is the manuscript technically sound, and do the data support the conclusions?

The manuscript must describe a technically sound piece of scientific research with data that supports the conclusions. Experiments must have been conducted rigorously, with appropriate controls, replication, and sample sizes. The conclusions must be drawn appropriately based on the data presented.

Reviewer #1: Yes

2. Has the statistical analysis been performed appropriately and rigorously?

Reviewer #1: Yes

3. Have the authors made all data underlying the findings in their manuscript fully available?

The [PLOS Data policy](http://www.plosone.org/static/policies.action#sharing) requires authors to make all data underlying the findings described in their manuscript fully available without restriction, with rare exception (please refer to the Data Availability Statement in the manuscript PDF file). The data should be provided as part of the manuscript or its supporting information, or deposited to a public repository. For example, in addition to summary statistics, the data points behind means, medians and variance measures should be available. If there are restrictions on publicly sharing data—e.g. participant privacy or use of data from a third party—those must be specified.

Reviewer #1: No

**Thank you for the point. Please find the data availability statement in our manuscript as follows.**

**“Data availability statement**

**The data are owned by a third party and the authors do not have permission to share the data. Requesting access to The Guideline Advantage (TGA) data must be done by contacting the American Heart Association via email:** [**qualityresearch@heart.org**](mailto:qualityresearch@heart.org)**. The study team will be able to share the code related to the analyses at the time of acceptance of the manuscript.”**

4. Is the manuscript presented in an intelligible fashion and written in standard English?

PLOS ONE does not copyedit accepted manuscripts, so the language in submitted articles must be clear, correct, and unambiguous. Any typographical or grammatical errors should be corrected at revision, so please note any specific errors here.

Reviewer #1: Yes

5. Review Comments to the Author

Please use the space provided to explain your answers to the questions above. You may also include additional comments for the author, including concerns about dual publication, research ethics, or publication ethics. (Please upload your review as an attachment if it exceeds 20,000 characters)

Reviewer #1: The authors investigate time-series cardiovascular risk factors and the association with cancer screening.

Major

- Average AUC values are not listed in the result section. Please do so.

**Thank you for the great point. We have added the average AUC values in the result section.**

- Discussion, please do not repeat your introduction in the first paragraph but list your main findings instead.

**Thank you for the great point. We have modified the first paragraph by listing our main findings in discussion section.**

- The discussion is short and interpretation of results is limited. Please expand.

**Thank you for the great point. We have expanded the discussion section and interpreted more of the results.**

- AUC of models is quite limited, the authors should address this.

**Thank you, this is a great point. We have addressed this limit in the limitation section.**

- It should be recommended to post the deep learning protocol at repositories like github, for other users to test.

**Thank you for the great suggestion. We have created a github repositories and posted the related Python codes. Please find the link:** [**https://github.com/aixiaguo/Python-Code**](https://github.com/aixiaguo/Python-Code)

Minor

- Introduction is on the long side please reduce to a single page.

**Thank you, we reduced the introduction to one page.**

- Avoid speaking language like “Fortunately”

**Thank you, we have deleted the speaking language like “Fortunately”.**
