# Supplementary material for: Time-series cardiovascular risk factors and receipt of screening for breast, cervical, and colon cancer: The Guideline Advantage
Source: PLoS One. 2020 Aug 13;15(8):e0236836. doi: 10.1371/journal.pone.0236836 (PMC7425922; doi:10.1371/journal.pone.0236836)
Supplement: Attachment — Submitted filename: Response to Reviewers.docx [file pone.0236836.s002.docx]

**Response to reviewer comments**

We would like to thank reviewers for the helpful comments. Please find our responses to the reviews below in bold. We hope you find the revised manuscript suitable for publication.

Reviewers' comments:

Reviewer's Responses to Questions

**Comments to the Author**

1. If the authors have adequately addressed your comments raised in a previous round of review and you feel that this manuscript is now acceptable for publication, you may indicate that here to bypass the “Comments to the Author” section, enter your conflict of interest statement in the “Confidential to Editor” section, and submit your "Accept" recommendation.

Reviewer #1: All comments have been addressed

2. Is the manuscript technically sound, and do the data support the conclusions?

The manuscript must describe a technically sound piece of scientific research with data that supports the conclusions. Experiments must have been conducted rigorously, with appropriate controls, replication, and sample sizes. The conclusions must be drawn appropriately based on the data presented.

Reviewer #1: Yes

3. Has the statistical analysis been performed appropriately and rigorously?

Reviewer #1: Yes

4. Have the authors made all data underlying the findings in their manuscript fully available?

The [PLOS Data policy](http://www.plosone.org/static/policies.action#sharing) requires authors to make all data underlying the findings described in their manuscript fully available without restriction, with rare exception (please refer to the Data Availability Statement in the manuscript PDF file). The data should be provided as part of the manuscript or its supporting information, or deposited to a public repository. For example, in addition to summary statistics, the data points behind means, medians and variance measures should be available. If there are restrictions on publicly sharing data—e.g. participant privacy or use of data from a third party—those must be specified.

Reviewer #1: No

**Thank you. As the datasets are from a third party, we do not have permission to share the data. We stated this in the Data availability statement as follows.**

**“Data availability statement**

**The data are owned by a third party and the authors do not have permission to share the data. Requesting access to The Guideline Advantage (TGA) data must be done by contacting the American Heart Association via email:** [**qualityresearch@heart.org**](mailto:qualityresearch@heart.org)**. The study team will be able to share the code related to the analyses at the time of acceptance of the manuscript.”**

5. Is the manuscript presented in an intelligible fashion and written in standard English?

PLOS ONE does not copyedit accepted manuscripts, so the language in submitted articles must be clear, correct, and unambiguous. Any typographical or grammatical errors should be corrected at revision, so please note any specific errors here.

Reviewer #1: Yes

6. Review Comments to the Author

Please use the space provided to explain your answers to the questions above. You may also include additional comments for the author, including concerns about dual publication, research ethics, or publication ethics. (Please upload your review as an attachment if it exceeds 20,000 characters)

Reviewer #1: The authors addressed most comments to satisfaction. One minor comment:

Please start discussion listing the most important finding: “In this study, we demonstrated associations between time-series”, “Regular cancer … after cancer screenings” can be deleted.

**Thank you. We really appreciate your great comments.**

**We have deleted “Regular cancer … after cancer screenings”.**
